# The Impact of the Yeoh Model’s Variability in Contact on Knee Joint Mechanics

**DOI:** 10.3390/ma18030576

**Published:** 2025-01-27

**Authors:** Łukasz Andrzej Mazurkiewicz, Adam Ciszkiewicz, Jerzy Małachowski

**Affiliations:** 1 Faculty of Mechanical Engineering, Military University of Technology, 00-908 Warsaw, Poland; lukasz.mazurkiewicz@wat.edu.pl; 2 Faculty of Mechanical Engineering, Cracow University of Technology, 31-155 Cracow, Poland; adam.ciszkiewicz@pk.edu.pl

**Keywords:** cartilage material properties, hybrid modeling, Sobol sequence, uncertainty, ligament, finite element, multibody

## Abstract

The aim of this study was to assess the impact of the variability of the Yeoh model when modeling the contact of bones through cartilage in the knee in compression and flexion–extension within a hybrid knee model. Firstly, a Sobol sequence of 64 samples and four variables representing the Yeoh parameters of the cartilage of the femur and tibia was generated. Based on these samples, 2 × 64 finite element contact models of the geometry of the sphere plane were generated and solved for healthy tissue affected by osteoarthritis. The resulting indentation curves were incorporated into a multibody knee joint model. The obtained results suggested that cartilage variability severely affected the knee in compression by up to 32%. However, the same variability also affected the flexion–extension motion, although to a lesser extent, with a relative change to the range of angular displacements of almost 7%. Osteoarthritic tissue was consistently more affected by this variability, suggesting that when modeling degenerated tissue, complex joint models are necessary.

## 1. Introduction

The knee joint is often considered one of the most complex joints in the body [1] and is also frequently injured [2]. To improve the quality of life of patients recovering from injuries, as well as the safety and success rates of the surgical procedures, validated and trustworthy models of the joint are necessary. However, body joints are often composed of multiple elements with a nonlinear material response that experience large displacements, which makes modeling a challenge. The typical elements of the synovial joints, such as the knee, include ligaments for transferring tensile loads and the bone–cartilage interface for compressive loads. The contact of the articulating surfaces is realized through at least two layers: the bone and the cartilage. At this interface, the bone functions as a stiff basis; in some of the models, it is assumed to be rigid [3,4,5]. More detailed studies employ linear isotropic materials to represent its behavior [6]. It is worth mentioning that the bone itself has a layered structure [7]. On the other hand, cartilage is mostly made up of water and collagen [8]. It is a soft tissue with a nonlinear material response [9,10], providing a lubricated surface in the joint [8]. The meniscus is an additional soft tissue element in the joint responsible for the absorption of shocks and the distribution of loads [11]. In some knee models, it is omitted [12,13]. These elements create a complex contact interface that can be challenging to properly reproduce in a model.

A common solution to this problem is to treat the interacting bodies as rigid and describe the contact problem with constraint equations. These implementations range from simplified representations of kinematic pairs [14,15,16,17] to complex formulations featuring multiple contact pairs, corresponding to the anatomical characteristics of the bones [18,19,20,21,22]. Their main advantage lies in a low number of parameters and low computational complexity. However, the material behavior of the articulating bodies is not explicitly defined in these approaches, limiting their application to cartilage-related studies. Another efficient option is deformable contact pairs within the multibody framework. These are typically formed in the Hertzian formulation, as seen in [12,13,23], and do not replicate the nonlinear characteristics of cartilage and the layered structure of the bone–cartilage interface. While the finite element (FE) method [3,6,24,25,26,27] provides a more accurate way to model the bone–cartilage interface, it is also more expensive numerically. However, this approach can be combined with the multibody framework to create an efficient hybrid with physical contact with the articular [24].

Regardless of the assumed modeling approach, the joint models feature a large number of uncertain parameters. Accounting for this parameter variability is challenging and requires specialized methods. One of the most common approaches is to sample the model multiple times. Sampling can be carried out by changing only one parameter at a time [4,28,29,30,31], which is often referred to as a sensitivity analysis. However, for models with complex behavior, sampling is typically performed on all the parameters at the same time. These approaches include the global sensitivity [32] and the screening method [18]. To lower the required number of samples, i.e., model solutions, specialized sequences can be used. In this case, the common choices include the Sobol sequence [33] and Latin hypercube sampling [34].

Parameter variability is an important topic in knee joint modeling and lower limb modeling in general. A large number of studies investigated the variability in musculoskeletal modeling [18,32,35,36,37,38,39]. This typically included analyzing the effect of the variability on the kinematics, contact forces or muscle forces. As the models were formulated under a multibody framework and mainly utilized geometric constraints, the effect of the cartilage material variability was not directly assessed. The effect of cartilage degeneration was addressed in [40] within a musculoskeletal model, but with geometric changes to the model rather than material variability. On the other hand, Ref. [12] directly analyzed the effect of the contact formulation on the motion and forces within a dynamic multibody model of the knee. However, the variability of the cartilage material was not addressed. Furthermore, the cartilage was simplified with Hertzian contact pairs. This material variability was analyzed in [24,41], but again with simplified Hertzian pairs or a hybrid model, but with linearized cartilage behavior. The variability analysis was also performed for complex FE models [4,9,42,43,44]. A sensitivity study [4] only considered the contact formulation and the material variability was not included. Study [42] focused on the effect of ACL reconstruction on the stress in the cartilage. Paper [43] focused on development of a statistical FE model for the knee, taking into account the shape variability of the bones and the cartilage, while [44] analyzed the variability within a knee simulator experiment. The cartilage material variability was directly analyzed using the FE method [9]. The geometry of the samples was simplified into a cuboid to reflect the experimental conditions. Experimental studies on the cartilage variability of the bone surfaces were also performed [45,46].

While the aforementioned studies represent a wide range of variability assessments for knee modeling, none of them focused directly on analyzing the nonlinear cartilage material variability and its effect on the ligament forces within the passive motion of the knee and considering the changes caused by osteoarthritis (OA). This issue is significant as cartilage is one of the most important elements of the knee, crucial for its functioning. Its degeneration is rapidly becoming a societal problem that affects up to 15% of people [47]. Therefore, the motivation for this study is to provide fast tools to understand the effect of cartilage properties on daily activities, such as standing and walking, and analyze this parameter variability within a realistic knee joint setting while accounting for both healthy and the OA-affected tissues.

In this study, the aim was to analyze the impact of contact material parameters on the knee joint through the use of the Sobol sequence and hybrid modeling. The research was based on and extended three published studies [9,13,24]. This study built on [9] by featuring a contact model with knee joint geometry, sampled using a quasi-Monte Carlo sequence of 64 samples, covering the parameter space more evenly. In addition, contact models were also incorporated and tested in a hybrid knee joint model in flexion and extension based on [13,24], which reflected realistic loading conditions for cartilage. Finally, this study extended an already published hybrid model [24] by featuring nonlinear cartilage material formulation. The assumed layered sphere–plane contact pair represents one of the simplest FE contact models with explicit cartilage layer definition. The symmetry of this model allowed for both easy integration into the multibody framework as well as fast simulation times in FE software. This meant that the FE contact model could be sampled multiple times at a low cost and incorporated into a multibody model, creating a very realistic fast hybrid. The low numerical complexity of the hybrid helped to generalize the results in the second stage of sampling performed using the hybrid. This allowed us to test 32 variants of the ligament system with 64 FE contact variants, creating an extensive database of 4096 unique hybrid knee models in dynamic flexion–extension under cartilage material variability. All the hybrids were solved in less than 40 min on consumer-grade hardware. When compared to the previously published studies on knee joint variability [4,9,12,18,24,32,35,36,37,38,39,40,41,42,43,44], this research presents a unique and detailed analysis of the effect of cartilage material variability on the passive motion of the knee and ligaments and the contact loads for both healthy and OA-affected cartilage. The cartilage variability was isolated from other parameters and tested using multiple variants of the knee model, which was made possible by the low numerical complexity of the proposed hybrid approach. Since the hybrid model is purely driven by load and contains no rigid constraints, it could be used to analyze how cartilage variability affects the loads transferred by the ligaments and provide novel insights into how this load distribution changes with cartilage degeneration. To our knowledge, such an extensive cartilage material variability analysis was not performed before.

The main objective of this study was to evaluate the impact of the variability of the Yeoh constitutive model when modeling articular contact in the knee. The cartilage was analyzed in both compression and flexion–extension of the hybrid knee joint model. To do so, a Sobol sequence of 64 samples and four variables was generated. Its variables represented the Yeoh parameters of cartilage of the femur and tibia assumed after [9]. Using these samples, 2 × 64 finite element contact models of the geometry were generated and solved for both healthy tissue and OA. The resulting indentation curves were incorporated into 32 variants of a multibody knee joint model, creating 4096 realizations of the hybrid model.

The results obtained suggested that cartilage variability severely affected the knee in compression by up to 32%. However, the same variability also affected the flexion–extension motion, although to a lesser extent, with relative change to the range of angular displacements of almost 7%. Osteoarthritic tissue was consistently more affected by this variability, suggesting that when modeling degenerated tissue, complex joint models are necessary.

## 2. Materials and Methods

**Figure 1:**
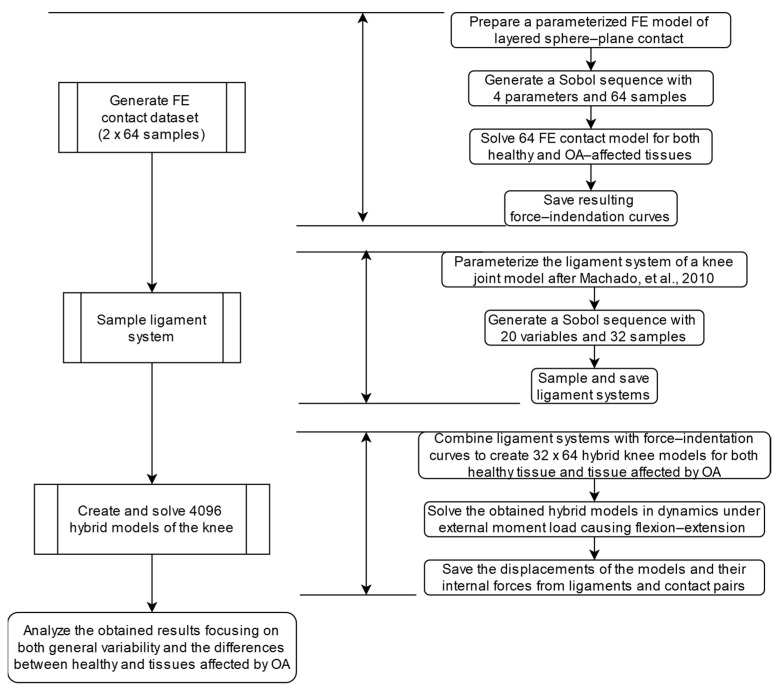
A schematic overview of the present study [13].

As mentioned above, the main objective of this study was to analyze the effect of the Yeoh variability on knee joint mechanics. A schematic overview of this study is presented in the form of a block diagram in Figure 1. Firstly, the contact interface in the knee was modeled as a layered sphere–plane contact pair with a nonlinear material formulation for cartilage solved by indenting the sphere into the plane in multiple FE simulations based on a Sobol sequence with 64 samples for healthy and OA tissue. The resulting force–indentation curves were then incorporated into a multibody knee model to assess the Yeoh variability within the flexion–extension motion of the knee. The results obtained were then analyzed using common statistics, including the mean, standard deviation, and coefficient of variation. The following subsections detail each part of this study.

### 2.1. Developing the Contact Model

The contact model for the knee joint was made to directly reflect that of a knee joint model proposed in [13]. This meant that the knee contact interface was simplified to sphere–plane interaction for each condyle, with the sphere and plane corresponding to the femur and tibia, respectively. The radius of the sphere was set at 40 mm. In contrast to the original [13], both interacting bodies had a two-layer structure, with the inner part substituting the bone with a linear isotropic material and the outer part modeling the cartilage using the Yeoh nonlinear constitutive law. The usage of hyperelastic models such as the Ogden, Mooney–Rivlin, neo-Hookean or Yeoh model introduces the nonlinear behavior of tissue and this approach is often used in knee models [48,49]. More advanced models that take into account anisotropy or the strain rate dependency can also be found [10,50], but their large numbers of parameters may result in the variability analysis of such models requiring very large computational resources. Furthermore, experimental tests of cartilage [51] confirmed that the assumption of incompressibility is acceptable and this simplification is widely used [52,53].

**Figure 2:**
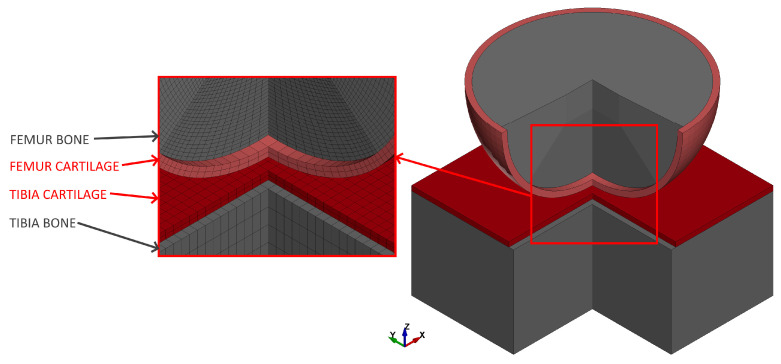
The FE contact model with the cartilage parts: the Yeoh model’s variable parameters.

The incompressible behavior of the material was described using the second-order strain energy function as follows:

(1) $$W=C_{10}\left({\bar{I}}_{1}-3\right)+C_{20}{\left({\bar{I}}_{1}-3\right)}^{2},$$

where $C_{10}$ and $C_{20}$ are independent material constants, ${\bar{I}}_{1}=\lambda_{1}^{2}+\lambda_{2}^{2}+\lambda_{3}^{2}$ is the first deviatoric invariant of the right Cauchy–Green deformation tensor and $\lambda_{i}$ are the principal stretches. The cartilage thickness for both bones was assumed after an experimental study [9] and was equal to 2.1 mm for the tibia and 2.3 mm for the femur. Previous studies [24] showed that bone stiffness had a negligible impact on the contact interaction. Therefore, the bone material for both bones was the same, with a Young’s modulus of E_b_ = 17,200 MPa and a Poisson’s coefficient of 0.39, assumed after [12]. The cartilage material differed for both, as seen in Figure 2.

The model was made with a parametric LS-Prepost 4.9 script and solved with LS-Dyna R13, a commonly used commercial FE software. Selectively reduced, fully integrated brick elements with 8 integration points were used to model the bodies. The cartilage layer and bone elements were connected by common nodes. The average edge length of the element was 2 mm in the contact area, with two elements that form the cartilage layer through its thickness.

The solution was obtained using a modified Newton–Raphson static integration scheme. The displacement (the current step, despite the total displacement-based norm) and the relative convergence tolerance of the residual force were equal to 1 × 10^−4^. The relative energy tolerance was set to 1 × 10^−7^ to improve the accuracy of the calculations.

The penalty-based segment–segment-based nonlinear contact algorithm was used in the FE solution. Additionally, to improve the accuracy of the force indentation response, the stiffness of the contact interface was increased by the scaling factor Sf = 50. The mesh size and the contact stiffness parameter were determined in the sensitivity study. The total number of elements was 1,220,000.

The loading conditions corresponded to the indentation of the sphere in the plane, so the prescribed displacement was applied to the nodes of the wall of the upper hemisphere. The sphere was indented into the plane up to 1.00 mm in 20 steps, recording the force required to achieve each indentation. The force–indentation curve was the output of the model. The computational time was between 8 and 10 min using 6 cores of an i9-12900K CPU by Intel, Santa Clara, CA, USA for one set of parameters.

### 2.2. Designing the Sample Sequence

The aforementioned FE contact model was parametrized with four variables for the variability analysis of the Yeoh cartilage model. These variables represented the Yeoh constants C_10_ and C_20_ for the cartilage of both the tibia and the femur (see Equation (1)) and were scaled to vary in the range of the mean ± 1 standard deviation assumed after [9], see Table 1.

To reduce the numerical complexity of the experiment, the sampling sequence was based on Sobol [33] and contained 64 samples for the four aforementioned variables. The sequence was generated in Scipy 1.12.0 [54]. Then, LS-Prepost was used to create 64 variants of the contact model based on the sequence. These LS-Dyna simulations were performed for both healthy and OA tissue [9], resulting in 2 × 64 (128) variants of the FE contact model. The force–indentation curve was recorded for each FE variant. Linear interpolation and extrapolation were used to make the curve continuous.

### 2.3. Hybridizing the Knee Model

**Figure 3:**
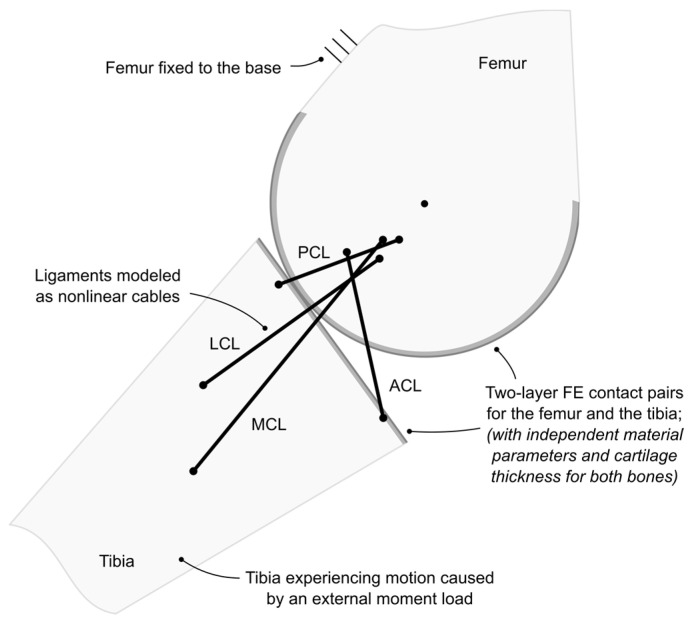
A schematic drawing of the hybrid model of the knee joint in the sagittal plane with parameters based on [13], except for the cartilage material parameters, which were based on [9]. The model is at around 55 degrees of flexion, which was the starting point for both flexion and extension.

The next step involved integrating the resulting force–indentation curves from the 128 FE simulations into a planar knee joint model assumed after [13] by replacing the existing Hertzian contact pairs. The resulting hybrid knee model (Figure 3) focused on the tibiofemoral part of the joint and featured four nonlinear cables substituting for ligaments and FE sphere–plane contact for bone contact through cartilage. The ligaments included the anterior cruciate ligament (ACL), posterior cruciate ligament (PCL), medial collateral ligament (MCL) and lateral collateral ligament (LCL).

The hybrid model was implemented in Python and featured dynamics solved with the semi-implicit Euler method and a step of 0.001 s. The model was simulated in flexion and extension with an external moment ranging from 0.0 Nm to 3.5 Nm for extension and from 0.0 Nm to −3.5 Nm for flexion. Both simulations lasted 16.0 s and started at the neutral configuration of the model, around 55 degrees flexion. The external moments were applied in five steps, while their values were selected so that the indentation of the contact pairs did not significantly exceed 1.0 mm in the flexion–extension—the limit in FE simulations. Additional global damping was also used to allow the model to reach equilibrium for any loading condition within the prescribed simulation time. Finally, the results were converted to the angle domain of the knee joint angle θ, which is the preferred method for presenting knee joint data.

### 2.4. Generalizing the Results from the Hybrid Model with the Second Stage of Sampling

As mentioned above, the main novelty and the advantage of the proposed approach lie in the development of a hybrid knee framework for use in efficient variability analysis, modeling the knee within a multibody framework and using FE contact pairs with an explicit cartilage layer. The hybrid model obtained was very efficient numerically and allowed for a thorough assessment of the cartilage material variability.

The variability of parameters is acknowledged in biomechanical modeling. While the topic of this study is cartilage material variability, the same variability affects knee ligaments. In fact, the subjective nature of geometric parameters can lead to variability in acquisition of up to ±2.5 mm [55]. Therefore, to generalize the results obtained in this study, the resulting hybrid models were sampled once more, creating 32 unique ligament system variants, each solved with 64 FE contact variants for both the healthy and OA-affected groups, which resulted in a total of 4096 unique hybrid model variants. This was achieved by generating a second Sobol sequence, this time 20-dimensional and composed of 32 samples. This sequence defined the changes in the ligament attachments to the tibia and femur (four ligaments × two attachments × two coordinates = 16 parameters) and their corresponding stiffness values. The samples obtained were scaled to ±1.0 mm and ±10% for geometry and stiffness, respectively. These values were within the ranges commonly reported and used for ligament variability [23,32,41,55]. The 20-parameter sample was then combined with every one of the 64 FE contact variants, resulting in 32 × 64 = 2048 unique models for both the healthy and OA-affected groups. This created a very large database of paired hybrid models to properly assess the effect of cartilage variability on. The time required to solve this large batch of 4096 models was only 36 min using 15 threads of Ryzen 6800H by AMD, Santa Clara, CA, USA in Python.

### 2.5. Assessing the Results

As mentioned above, the aim of this paper was to quantify the effect of the variability of the Yeoh model on cartilage in a realistic setting of a hybrid knee joint model. The analysis was performed in two steps. First, the effect of the variability was accounted for only in compression. This reflected weight-bearing conditions, such as standing. Then, the variability in knee flexion–extension under external moment loads was tested using the proposed hybrid models. To quantify the effect of the Yeoh variability and not the entire model, the models were analyzed in batches of 64 per healthy and OA-affected group for each of the 32 ligament system variants. This allowed us to isolate the effect of cartilage variability from ligament variability while creating a large pool of reference hybrid models. The main point of the evaluation was to evaluate the values of the contact forces generated within these two scenarios. For flexion–extension, an additional assessment of the angular displacements and ligament forces was performed. The results were divided into the healthy and OA-affected subgroups and analyzed using typical statistical quantities—mean, standard deviation, and coefficient of variation (CV), which is the ratio between the standard deviation (SD) and the mean. For angular displacements, an additional metric of the relative change of displacement *RDiff* was used and computed for 64 hybrid models within each of the 32 variants as follows:

(2) $${RDiff}_{i}=\frac{{range}_{max,i}-{range}_{min,i}}{{range}_{mean,i}}\cdot 100\%,$$

where *RDiff_i_*—the relative change of angular displacement between the 64 models for a specific variant and for group *i*, with *i* being either the healthy, OA-affected or pooled (combined) group; and *range_max,i_*/*range_min,i_*/*range_mean,i_*—the max/min/mean range of the angular displacements within the 64 hybrid models of a specific model variant for group *i*.

## 3. Results

The results section is subdivided into two parts. The first details the results of the compression test based on the 64 variants of the FE contact pair in both the healthy and the OA-affected groups. The second focuses on the flexion–extension motion in the hybrid knee joint model.

### 3.1. Compressive Loads in the Contact Pair

**Figure 4:**
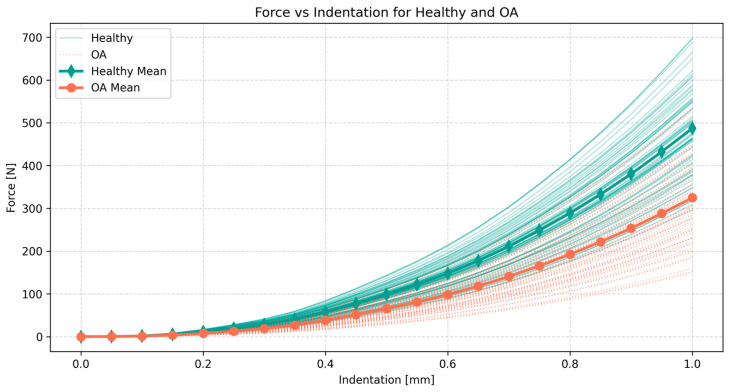
The contact force versus the indentation in 64 variants and the mean of the FE model of the sphere–plane layered contact pair in the knee with the Yeoh model for cartilage, accounting for its variability in both healthy and OA-affected tissues.

The compression curves for the knee were obtained from the FE contact model described in Section 2.1 and Section 2.2. This model was solved in 64 variants based on the Sobol sequence for the healthy and OA groups. The resulting 128 force–indentation curves for 20 values of indentation of up to 1.00 mm are presented in Figure 4.

Both groups exhibited visible variability in their force curves, with standard deviations of approximately 101 N and 98 N for the healthy and OA groups, respectively, under indentation of 1.00 mm. However, the mean force at this indentation was higher for the healthy group at 487 N compared to the OA group at 325 N. These different mean values resulted in the CV being around 21% and 30% for the healthy and OA groups, respectively, indicating the greater relative variability of the contact force within the contact pairs affected by OA. With the combined groups, the mean value was 406 N, with a standard deviation of 129 N, resulting in the coefficient of variation standing at 32%. The lowest recorded force at 1.00 mm was 129 N, while the highest peaked at 698 N, illustrating the high variability of the contact force in compressive loads on the contact pairs in the knee joint.

### 3.2. External Moment Causing Flexion–Extension in the Hybrid Knee Models

**Figure 5:**
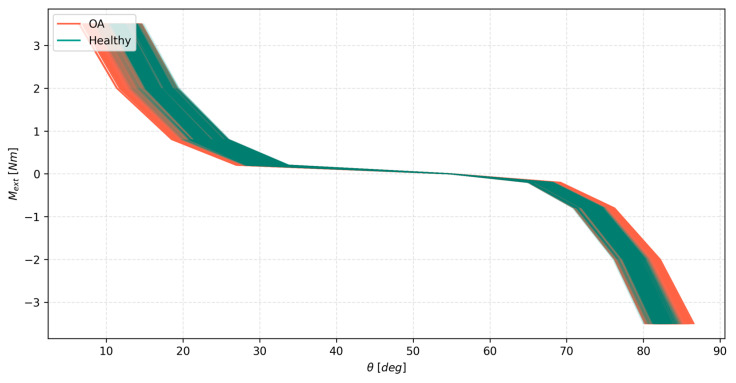
The external moment *M_ext_* as a function of the angle of the knee joint θ, with 0 deg corresponding to a fully extended knee for both the healthy and OA tissue groups, for all 32 variants of the hybrid model.

**Figure 6:**
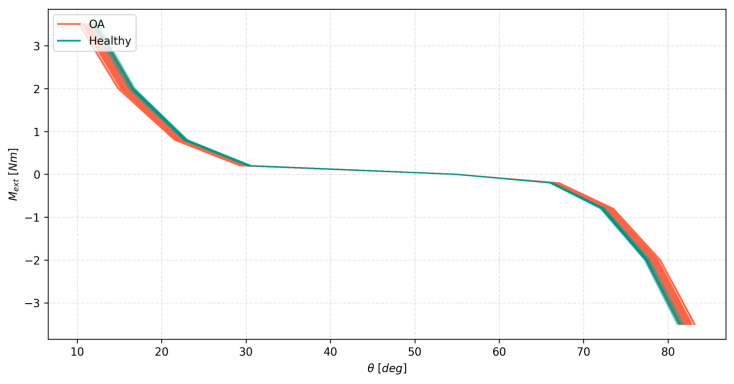
The external moment *M_ext_* as a function of the angle of the knee joint θ, with 0 deg corresponding to a fully extended knee for the healthy and OA tissue groups, first variant only for clarity.

As mentioned before, this second test involved 32 unique variants of the ligament system, each tested with 64 FE contact pairs. Each variant was analyzed in terms of the effect of cartilage variability on the range angular displacement, the ligament forces, and the contact forces in flexion–extension. The general effect of cartilage variability on the angular displacements in all the variants is presented in Figure 5. Two observations can be made from it. Firstly, OA-affected tissue resulted in more compliant knee joint models. Their cumulative displacement range was greater than that of healthy tissues. Secondly, the OA-affected models covered nearly the entire range of the combined groups. This meant that the general effect of cartilage variability was more pronounced for the models affected by OA. This is further evidenced in Table 2. The results showed that the relative range of model displacements within all the variants was almost twice as large for the OA group compared to the healthy group. Interestingly, these values were very consistent between the variants, as seen in Table 2. All this showed the variants being actually affected by the changes in the ligaments, which could be seen in the last column of Table 2 and in Figure 5. This means that the displacements of the OA model being more affected by the cartilage variability is more of a general trend rather than a specific result valid for one model only. In terms of the actual values, the coefficient of variation remained at a relatively low level of 1.47% when considering the combined pool of OA and healthy models. However, the relative change to the ranges averaged 6.73%, which is a large portion of the range of displacements of the model, suggesting that cartilage variability does, in fact, affect the motion of flexion–extension in a meaningful way. This was further visualized in one of the variants of the model, as seen in Figure 6. Again, the OA-affected group covered almost the entire range of the models. The healthy group provided a stronger load response.

**Figure 7:**
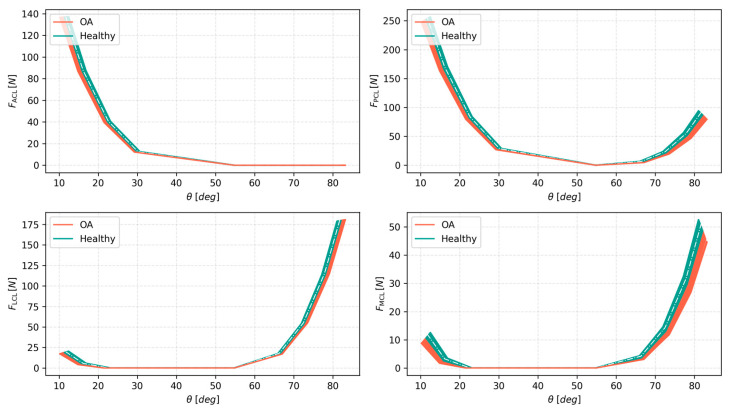
Forces generated by ligaments with respect to the knee joint angle θ under external moment loads of up to 3.5 Nm in both flexion and extension for the healthy and OA groups, only for the first variant of the hybrid model, for clarity. Healthy is drawn on top of OA, with an additional white line representing the last solution in the OA group, to showcase that OA covers most of the solution ranges.

**Figure 8:**
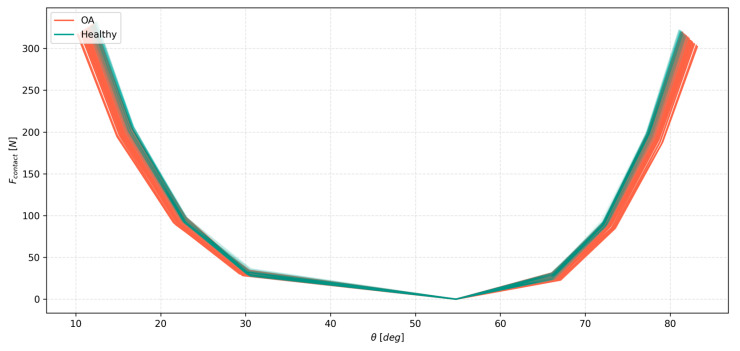
Forces generated by the contact pairs with respect to the angle of the knee joint angle θ under external moment loads of up to 3.5 Nm in both flexion and extension for the healthy and OA groups, for one selected variant of the hybrid model only, for clarity. Healthy is drawn on top of OA.

The angular displacements of the joint paint only part of the picture. To fully analyze the situation, a similar analysis was performed for the ligament and contact forces. Table 3 presents a general summary of the coefficients of variation for all the ligament forces in all the studied variants of the ligament system, while Figure 7 and Figure 8 present the actual forces in one of the variants for clarity.

This time, the results were even more intriguing. The variability of cartilage affected the different ligaments in unique ways. As seen in Table 3 and Figure 7, the ACL remained indifferent to the cartilage material, with an average CV in extension of less than 0.1%, while being entirely inactive in flexion. A similar situation was observed for the LCL in extension. However, the remaining ligaments, the PCL and MCL, were affected. These changes were not as pronounced as the change in the contact force in compression; nevertheless, the CV for the MCL affected by OA averaged just less than 4% for flexion while generating forces close to 60 N. In contrast, the PCL’s averaged CV in the OA group was 4.61%, with a peak value of over 7%. Again, the maximum values were obtained within the OA group.

The contact force in flexion–extension exhibited a generally much higher value than that of the ligament, as seen in Figure 8. While the peak values were affected by the cartilage variability in nominal values, their relative metrics, such as the CV, remained under 2% due to the high force values. Interestingly, the changes to the angular displacements were notable. The results showed that the hybrid model with tissue affected by OA visibly complained more than its healthy counterpart. These findings are substantial and might suggest compensation mechanisms within the joint with the degenerated cartilage. The CV was consistently higher within the OA-affected group.

## 4. Discussion

### 4.1. Compressive Loads

In terms of compression loads only, the variability of the Yeoh model for cartilage had a large effect on the models in both the healthy and OA groups. This was reflected in the large value of the CV, up to 32% for both groups combined. The relative variability was greater within the OA group, which also showed a lower mean value for the contact force. The lower contact force could be related to the inability of the OA tissue to withstand compressive loads, which is one of the primary functions of the knee.

The healthy tissue generally showed a steeper increase in stiffness, reaching forces up to 50% higher on average compared to those affected by OA. In the extreme cases, the model at the upper end of the healthy spectrum achieved the maximal force of the variant affected by OA at the lower end of around half of its indentation. This variability in compression output was generally in line with [9], where it was analyzed in samples with plug-shaped geometry compared to the more realistic contact pairs that represent idealized contact in the knee.

### 4.2. Flexion–Extension of the Knee

The cartilage variability also affected the hybrid model in flexion and extension. The primary finding in this case was the fact that the group affected by OA was consistently more affected than the healthy tissue, almost twice as much, as measured by the relative ranges and CV. The relative difference in the angular displacements was nearly 6% on average, whereas the healthy tissue averaged close to 3%. This finding was only achievable with the proposed method, as its low numerical complexity combined with an explicit cartilage layer facilitated such an extensive analysis of multiple variants of the knee model. In contrast, most quick models available do not have an explicit cartilage layer [16,19,20,45,46] or their model behavior description is significantly simplified [12,13,44]. This finding suggested that it might be possible to model the knee without explicitly considering the cartilage for healthy tissues, as the tissue and the resulting model are stiffer and more resilient to changes in the parameter of the cartilage material. However, when modeling degenerated tissue, the cartilage material effect cannot be omitted.

Analyzing the results in detail showed interesting internal behaviors, with different ligaments having distinct sensitivity to cartilage variability, and some of them, like the ACL, even being indifferent to it. Higher variability was consistently observed for tissue affected by OA. Furthermore, while the angular displacements between the variants were affected in similar ways, the same cannot be said for the ligament forces. In this case, the variation of the CV within the model variants might potentially suggest different compensation mechanisms present mostly in the OA-affected models.

Although cartilage variability had an effect on the hybrid knee model in flexion–extension, this effect was less pronounced than in compression. This could be partially explained by the fact that most of the range of motion in flexion–extension is achieved with the model loaded with only a minimal external moment. This range represents the low-stiffness zone of the knee. As seen in Figure 7 and Figure 8, the effect of variability becomes more pronounced for the higher values of the external moment. Another possible explanation is based on the fact that the stiffest FE contact model achieved the maximal force from the most compliant one in only around 0.5 mm less indentation. In the full knee model featuring the ligaments, the effect of a 0.5 mm change in the indentation might be even less, as the ligaments are generally not perpendicular to the tibial plane in the model. This, combined with the low ligament loads during this motion, likely made them largely indifferent to the variability of the contact material.

On the basis of the results obtained, it can be seen that the knee motions are affected in different ways by the material parameters than by the others, with flexion–extension and compression serving as the studied examples. Although these results are related to the selected knee model and could change with different implementations, they highlight the need to quantify the variability of the material parameters under varied loading conditions. It also emphasizes the need to account for variability when modeling tissue affected by OA, in which its effect was found to be greater.

### 4.3. Model Sampling to Generalize Results

With regards to the employed procedure for analyzing variability, it should be noted that the combination of two-step model sampling with the extendable Sobol sequence created a unique combination, taking advantage of a relatively small number of precomputed FE contact simulations to create a very extensive database of functional hybrid models of the knee. The low numerical complexity was one of its major advantages, requiring under 40 min to compute 4096 hybrid models in dynamic flexion–extension on consumer-grade hardware. This number could be scaled up if needed. This hybrid approach allowed for an explicit and physical definition of the cartilage layer, which is very difficult to achieve with the regular multibody method. As mentioned above, most models either simplify the structure of the bone–cartilage interface, as seen in [13], or omit this layer entirely [56]. The existing FE models of the knee [4] are a potentially good solution, but their numerical complexity does not allow for extensive sampling. The number of required samples is typically large, with examples including more than 1000 with specialized methods [18] or 10,000 with quasi-Monte Carlo sequences, as seen in [32]. The proposed approach bridges the gap by allowing both explicit and nonlinear cartilage layers, along with large numbers of sampled models for variability analysis.

### 4.4. Limitations

This research focused on analyzing the variability of the cartilage model under two realistic loading conditions for the knee, reflecting both compression and flexion–extension. However, displacements outside of the sagittal plane were not studied, as they are outside the scope of the proposed hybrid knee model. Furthermore, the loads and displacements of the studied model, including indentation, reflect only a portion of the allowable loads and the entire range of motion of the knee.

The accounting for the material variability of cartilage was a deliberate choice that allowed the judge to judge its effect on the mechanics of the knee. However, it should be mentioned that the variability within the joint can also be considered for other material parameters and the geometry of the entire system. This was partially achieved with the variants of the ligament system, but the ligament variability was not directly analyzed in this study.

## 5. Conclusions

This article aimed to quantify the effect of variability on the nonlinear formulation of cartilage material within a hybrid knee modeling environment under compression and flexion–extension. This was performed with a unique combination of two-stage sampling using the Sobol sequence. In the first stage, FE contact models were sampled. This was followed by a sampling of the ligament system in the second stage. In total, 4096 unique hybrid models were created and analyzed under different loading conditions, reflecting common daily activities.

The results suggested that cartilage variability had an effect on the joint in both compression and flexion–extension. Although the effect on flexion–extension was less than on compression, the relative changes to the angular displacements could be nearly 7%, which constituted a large part of the motion. The effect was notably pronounced for the models affected by OA, which were generally more compliant, both in compression and in flexion–extension.

These results suggested that it might be possible to model the knee without explicitly considering the cartilage for healthy tissues, since the tissue and the resulting model are stiffer and more resilient to changes in the parameters of the cartilage material. However, the cartilage material effect cannot be omitted when modeling degenerated tissue.

## Figures and Tables

**Table 1:** Range of variation of the Yeoh material constants for healthy cartilage tissue and OA assumed after [9]. These constants represent the variables of the parametrized FE contact model.

Material Constant	Healthy Tissue		OA Tissue	
	Min [MPa]	Max [MPa]	Min [MPa]	Max [MPa]
Femoral *C*_10_	0.6	2.2	0.4	2.0
Femoral *C*_20_	0.0	7.3	0.3	4.1
Tibial *C*_10_	1.2	2.8	0.3	1.9
Tibial *C*_20_	1.8	7.2	1.8	3.6

**Table 2:** Selected results and statistics on the relative change of angular displacement (RDiff) as given by Equation (2), the coefficient of variation (CV) in the healthy (H), affected by OA (OA) and combined (C) groups for all the variants of the model given by ID, together with the average value (AVG) of the variants and an additional maximum range of angular displacement within the models of each variant to showcase the differences between them (number of lines limited to first and last three).

ID	RDiff_H_ [%]	CV_H_ [%]	RDiff_OA_ [%]	CV_OA_ [%]	RDiff_C_ [%]	CV_C_ [%]	Range_C_ [deg]
1	3.16	0.81	5.54	1.35	6.42	1.40	73.02
2	3.32	0.85	5.84	1.42	6.76	1.47	74.59
3	3.29	0.84	5.79	1.41	6.7	1.46	75.09
⋮	⋮	⋮	⋮	⋮	⋮	⋮	⋮
30	3.26	0.84	5.74	1.40	6.63	1.45	74.4
31	3.32	0.85	5.83	1.42	6.75	1.47	72.10
32	3.36	0.86	5.91	1.44	6.85	1.49	74.57
AVG	3.31	0.85	5.82	1.42	6.73	1.47	74.40

**Table 3:** Selected results and statistics on the coefficient of variation (CV) for the forces of four ligaments (ACL, PLC, LCL, MCL) in extension in both the healthy (H) and OA-affected (OA) groups for all the variants of the model given by ID, along with the average value (AVG) and standard deviation (SD) in the variants. The CV is computed based on the max flexion force for the PCL, LCL and MCL and the max extension force for the ACL due to the ACL being inactive in flexion within the simulations (number of lines limited to first and last three).

ID	CV _ACL,H_ [%]	CV _ACL,OA_ [%]	CV _PCL,H_ [%]	CV _PCL,OA_ [%]	CV _LCL,H_ [%]	CV _LCL,OA_ [%]	CV _MCL,H_ [%]	CV _MCL,OA_ [%]
1	0.05	0.06	2.06	3.57	0.15	0.26	2.06	3.51
2	0.04	0.05	2.56	4.46	0.16	0.29	2.21	3.77
3	0.03	0.05	2.11	3.63	0.09	0.18	2.65	4.57
⋮	⋮	⋮	⋮	⋮	⋮	⋮	⋮	⋮
30	0.02	0.03	3.79	6.64	0.14	0.24	2.47	4.18
31	0.17	0.28	2.70	4.69	0.20	0.37	1.64	2.78
32	0.04	0.07	2.90	5.04	0.18	0.33	2.51	4.27
AVG	0.07	0.11	2.64	4.61	0.15	0.28	2.23	3.81

## Data Availability

The original contributions presented in this study are included in the article. Further inquiries can be directed to the corresponding author.
